# Magnetic resonance imaging template to standardize reporting of anal fistulas

**DOI:** 10.1007/s10151-020-02384-6

**Published:** 2021-01-05

**Authors:** I. Sudoł-Szopińska, G. A. Santoro, M. Kołodziejczak, A. Wiaczek, U. Grossi

**Affiliations:** 1 Department of Radiology, National Institute of Geriatrics, Rheumatology and Rehabilitation, Warsaw, Poland; 2 Department of Diagnostic Imaging, Warsaw Medical University, Warsaw, Poland; 3 Tertiary Referral Pelvic Floor Center, 4th Division of General Surgery, Regional Hospital, University of Padua, Piazzale dell’Ospedale 1, 31100 Treviso, Italy; 4 Department of Proctology, St. Elizabeth Hospital, Warsaw, Poland; 5 National Bowel Research Centre, Queen Mary University of London, London, UK

**Keywords:** Anal fistulas, Anal fistula imaging, Endoanal ultrasonography, Pelvic magnetic resonance imaging

## Abstract

Anal fistula (AF) is a common referral to colorectal surgeons. Management remains challenging and sometimes controversial. Magnetic resonance imaging (MRI) is commonly performed in initial workup for AF. However, reports often lack key information for guiding treatment strategies. It has been shown that with structured radiology reports, there is less missing information. We present a structured MRI template report including 8 key descriptors of anal fistulas, whose effectiveness and acceptability are being assessed in a cross-sectional study (NCT04541238).

## Introduction

Management of anal fistulas (AF) can be challenging for colorectal surgeons. Although fistulotomy is considered the gold-standard treatment for simple fistulas, repeated procedures are often required in complex cases. A thorough characterization of AF by clinical examination and imaging is pivotal in selecting the most appropriate treatment [1]. Profound technical variations exist in the surgical management of AF, making it difficult to reproduce and compare treatment outcomes among centers [2].

Magnetic resonance imaging (MRI) and endoanal ultrasonography (EAUS) are the most frequently used diagnostic modalities for preoperative assessment and follow-up [3, 4]. MRI has a broad field of view that well characterizes both sphincter anatomy and the perianal/perirectal regions (i.e., ischiorectal fossae and supralevator space). Administration of intravenous contrast medium helps to discriminate between scars and recurrent AF. Nevertheless, AF are often incompletely characterized in MRI reports thus challenging decision-making processes. Structured radiology reports have been shown to reduce missing information [5]. We sought to improve MRI reporting by developing a structured template to include the presence of 8 key descriptors of AF.

## MRI scanning technique

### Technique for anal fistula imaging

Pelvic MRI for the diagnosis of AF is conducted with body matrix or endorectal coils. The latter, however, are seldom used in the current practice as they are poorly tolerated. Moreover, endorectal coils have a limited field of view that reduces their usefulness in the diagnosis of inflammatory lesions spreading beyond the sphincters.

MRI planes are determined along the long axis of the anal canal, which results in acquiring oblique, axial, and coronal planes. The sagittal fast spin-echo T2-weighted sequence is usually conducted initially to acquire proper orientation and visualize the entire pelvis and anal canal. On the basis of this plane, further scanning is planned, i.e., axial and coronal planes. The MRI protocol used by the authors is presented in Table 1.

**Table 1:** Magnetic resonance imaging protocol for anal fistula imaging

Parameters	T2 TSE	T2 TSE	T2 TSE	T2w TIRM	T2 TIRM	T1 TSE FS	T1 TSE FS CM
Imaging planes	Oblique axial	Sagittal	Oblique coronal	Oblique axial	Oblique coronal	Oblique axial	Oblique axial
TR/TE (msec)	3020/10	5010/100	3800/100	4190/60	5340/60	545/10	545/10
FOV (cm)	260	250	250	290	380	260	260
Section thickness (mm)	4	4	4	4	4	4	4
Intersection gap (mm)	0.8 × 0.8 × 4.0	0.8 × 0.8 × 4.0	0.8 × 0.8x4.0	0.9 × 0.9 × 4.0	1.2 × 1.2 × 4.0	1.0 × 1.0 × 4.0	1.0 × 1.0 × 4.0
Matrix	320 × 256	320 × 256	320 × 256	320 × 256	320 × 256	320 × 256	320 × 256
Averages	1	1	1	2	1	1	1

*FOV* field of view, *TIRM* turbo inversion recovery magnitude, *TSE* time spin echo

### MRI anatomy of the anal canal

The internal anal sphincter (IAS) and external anal sphincter (EAS), the levator ani muscle, including its lowest part (i.e., the puborectalis [PR] muscle) as well as the ischiorectal fossae and supralevator space are generally assessed in the axial plane and, additionally, in coronal and sagittal planes in various sequences. In many patients, the discrimination between individual sphincters in time spin-echo (TSE) T1-weighted and fat saturation (FS) T1-weighted sequences can be difficult. In short tau inversion recovery/turbo inversion recovery magnitude (STIR/TIRM) sequences, the EAS and PR are difficult to identify. Conversely, the sphincters are easily visible in T2-weighted images and on postcontrast FS T1-weighted images. The IAS has slightly higher signal intensity than the EAS and PR. The intersphincteric space, seen in FS T1- and T2-weighted images, produces a high signal. MRI is superior to EAUS in reproducing very good images of fat tissue in the ischiorectal fossae and supralevator space [2]. This directly translates into optimal characterization of all pathologies affecting these regions.

### MRI classification of anal fistulas

There are two basic AF classification systems: the Parks classification from 1976 [6] and the Morris MRI grading system from 2000 [7]. Both systems take into account the course of AF in relation to the anal sphincters.

The Parks classification distinguishes four types of fistulas based on their course in relation to the EAS:

- Intersphincteric AF accounts for 45% of tracts. It penetrates the IAS and runs in the intersphincteric space to its external perianal opening (although it can have a blind subcutaneous ending).
- Transsphincteric AF accounts for 30% of tracts. It penetrates the IAS and then EAS at various levels and runs through the ischiorectal fossa to its external skin opening (it can be blind and end subcutaneously or in the ischiorectal fossa).
- Suprasphincteric AF accounts for 20% of tracts. It penetrates the IAS. At first, it runs upwards in the intersphincteric space to the supralevator space, crosses the PR, and bends downwards in the ischiorectal fossa to terminate in its external perianal opening (it can be blind and end subcutaneously or in the ischiorectal fossa).
- Extrasphincteric AF accounts for 5% of tracts. It opens internally to the rectum (although it can be blind, i.e., does not penetrate the rectal wall) as a complication of pelvic inflammation, trauma or surgery. It has a peripheral course outside of the sphincters in the ischiorectal fossa down to its external skin opening (it can also end blindly subcutaneously or in the ischiorectal fossa).

The Morris classification extends the Parks classification to incorporate accompanying abscesses, usually residual or incompletely emptied, and extensions. It has five grades:

- Grade 1: Simple linear intersphincteric fistula (as above in the Parks classification).
- Grade 2: Intersphincteric fistula with intersphincteric abscess and secondary fistulous tract.
- Grade 3: Transsphincteric fistula (as above in the Parks classification).
- Grade 4: Transsphincteric fistula with an abscess and secondary tract within the ischioanal or ischiorectal fossa. Abscesses can develop at any part of the fistula or its extension, but below the levator ani level.
- Grade 5: Supralevator and translevator disease (incorporates suprasphincteric and extrasphincteric fistulas from the Parks classification), i.e., all fistulas above the levator ani.

It must be noted that neither classification system includes all key information that should be available in an MRI report [8]. Furthermore, in the Morris classification, Grade 5 incorporates two types of AF with different etiology requiring different surgical management. Moreover, the Morris classification does not include supralevator extensions of transsphincteric fistulas, which are encountered in clinical practice. The cross-sectional diameter of the fistula tracts is a further important element for decision making. Indeed, AFs with less than 5 mm in cross diameter, simple, complete, and with straight course are amenable for laser treatment [9].

**Fig. 1:**
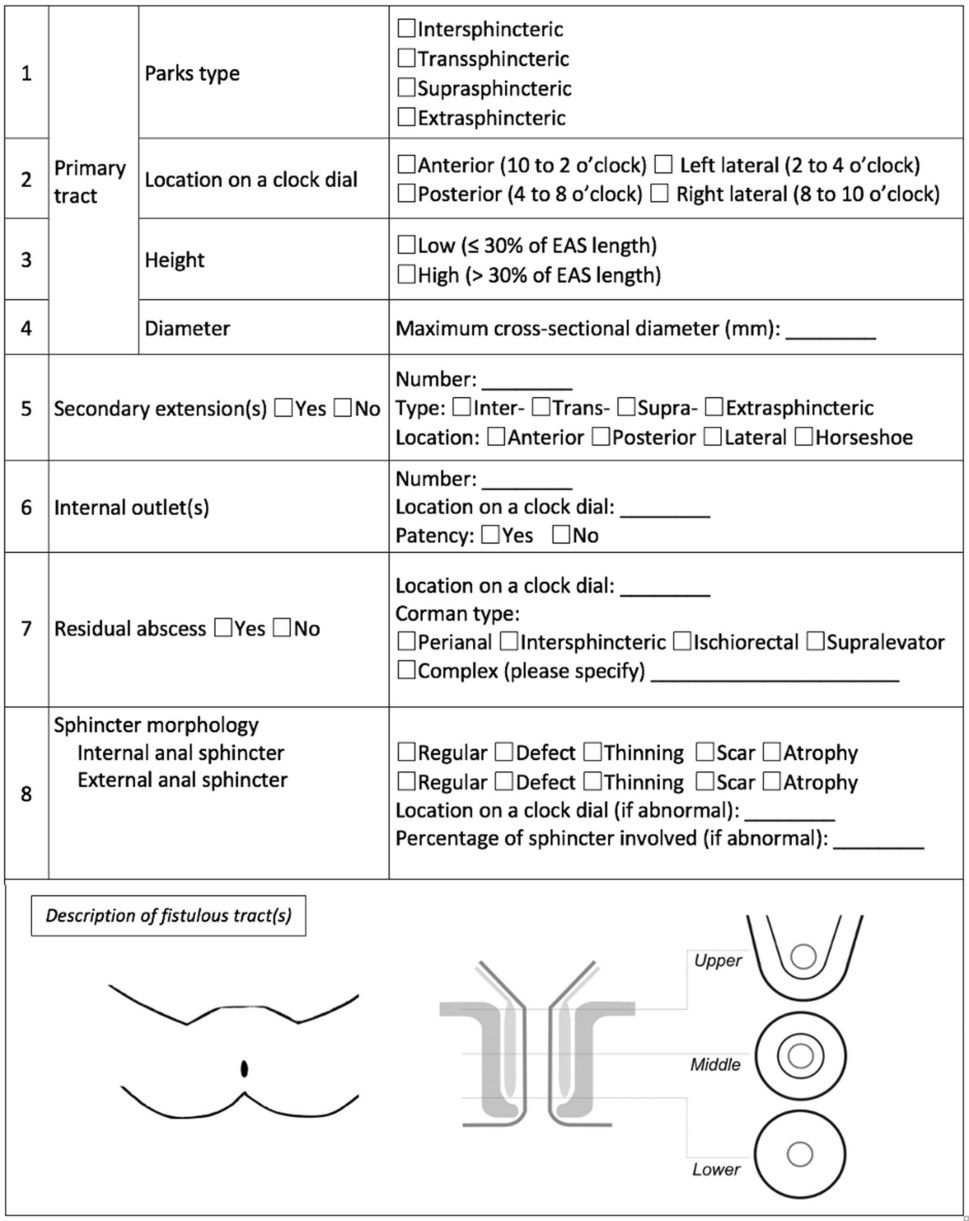
A novel magnetic resonance imaging template for a uniform description of anal fistula

Based on the above, we propose a novel MRI template for a uniform description of AF to include (Fig. 1):

1. Parks classification.
2. Radial localization in relation to the anal canal wall using a clock dial description, i.e., the specification at which hour a fistula crosses the EAS. The same principle is used in MRI, EAUS, and digital examination in the lithotomy position.
3. Height. This is of major importance for surgical planning. A low fistula traverses ≤ 1/3 of the EAS (i.e., the level at which only distal EAS is visible on axial MRI scans), whereas a high fistula traverses > 1/3 of the EAS (i.e., the level at which the IAS is visible medially to the EAS).
4. Cross-sectional diameter of the AF tract.
5. Description of any residual abscess, according to the Corman classification [10].
6. Description of any secondary extensions and determination of the number and location of branches. Secondary tracts are present in 5–15% of AF and may affect any level of the fistulous tract, but usually occur in the ischiorectal fossa, intersphincteric space and, more rarely, in the supralevator space. If the extension involves at least a half of the anal circumference (anteriorly, posteriorly, or laterally) is defined ‘horseshoe’ tract. These extensions are well visible in the axial planes.
7. Number, location, and patency of the internal opening. Information about the location (height and site based on a clock dial) of the internal opening is significant for the surgeon since failure of its removal will cause a recurrence. The assessment of patency or obstruction of the internal orifice is not always possible in MRI (nor it is in EAUS), which must be noted in an MRI scan report. The visualization of the external outlet is difficult on MRI and for this reason it is not included in the template.
8. Morphological condition of anal sphincters (A. regular; B. defect; C. thinning; D. scar; E. atrophy) with information concerning the level of the anal canal at which abnormalities are located as well as the clock dial description and percentage of sphincter circumference (size) involved.

In suspected postoperative recurrence, MRI is very helpful in differentiating active tract from inactive fistula and fibrotic scar which are hypointense in all sequences and do not undergo postcontrast enhancement.

The template does deliberately not include the following three types of AF due to their different etiology and management:

- Subcutaneous fistulas that in our practice are identified very rarely. Their etiology can be associated with purulent perianal conditions, which are not necessarily associated with anal crypt infection. In MRI, they are typically found medially to the IAS in the epithelial layer (i.e., do not penetrate the IAS) [11].
- Rectovaginal fistulas that usually develop due to an obstetric trauma. Axial and sagittal planes of contrast-enhanced MRI can detect even narrow (1–2 mm) fistulas, which are the most difficult to diagnose.
- Rectoperineal fistulas, with an etiology usually associated with perineal trauma or childbirth.

The proposed MRI template can also be used in Crohn’s disease to report a complete morphological image of the fistulous tracts. AF in Crohn’s are usually discussed separately due to their complex course, different etiology and therapeutic management. Such tracts do not originate from crypt infection but rather result from transmural spread of chronic granulomatous inflammation [12]. Approximately, 50% of these AF are high transsphincteric or high intersphincteric, and suprasphincteric tracts. They usually have horseshoe and supralevator extensions that make them likely to recur and challenging to treat. MRI may also visualize abnormal signals of the anal sphincters due to post-inflammatory changes associated to disorders of anorectal sensation or fecal incontinence. In patients treated with biological therapy, MRI is used for monitoring patient response to treatment.

## Conclusions

The proposed MRI template for the report of AF may be an effective and efficient way to improve characterization, direct management and thence appropriate follow-up of patients with AF, by making key descriptors available to surgeons. To test these hypotheses, we designed a study (NCT04541238) [13] to evaluate (1) the feasibility, acceptability, and effectiveness of the MRI template, (2) the reproducibility and the interobserver agreement in detecting AF descriptors, and (3) the efficacy of the template in enhancing the surgical decision planning as compared to standard MRI reporting. Based on the results of this study, the subsequent Step is to seek consensus among key opinion leaders in the field of surgery and radiology to confirm the minimum set of AF descriptors to be included in a synoptic MRI report.
